# An online taxonomic database of the stick insect (Phasmida) egg-parasitising subfamilies Amiseginae and Loboscelidiinae (Hymenoptera: Chrysididae)

**DOI:** 10.3897/BDJ.4.e7441

**Published:** 2016-01-08

**Authors:** Ed Baker

**Affiliations:** ‡ The Natural History Museum, London, United Kingdom

**Keywords:** taxonomic database, parasitoid wasp, Phasmida, Phasmatodea, stick insect, Hymenoptera, Chrysididae, Loboscelidiinae, Amiseginae

## Abstract

**Background:**

The wasp subfamilies Amiseginae and Loboscelidiinae (Hymenoptera: Chrysididae) were last catalogued in Kimsey and Bohart (1991). The subfamilies are considered to be obligate egg parasitoids of the Phasmida (stick insects), which are known to be pests in many areas of the world (Baker 2015). Our lack of knowledge of these wasps, in particular their host associations and host specificity, prevents studies into using them as potential control agents for pest phasmids. Phasmids are popular throughout the world with people from a wide range of backgrounds, from pet keepers to professional entomologists.

**New information:**

A taxonomic database of the subfamilies Amiseginae and Loboscelidiinae has been created as the Chrysididae SpeciesFile, summarising the current state of scientific knowledge about these groups. In addition, a bibliography of works on these subfamilies has been created. In total information is provided for 187 valid species.

## Introduction

The Phasmida Species File Online (Brock 2015) has become the defacto online resource for reliable taxonomic information on that order. It is hoped that a creation of a similar database of their predominant egg parasitoids will facilitate new research in this field. The host associations of the Amiseginae and Loboscelidiinae are poorly known. Only 12 unique associations at the species level have been recovered from the literature while developing the current project.

The Phasmida (= Phasmatodea; Phasmatoptera; Cheleutoptera) are large, obligate herbivores (Bedford 1978) that are known as pests of agriculture and forestry in North America, Asia, and Oceania (Baker 2015). The wasp subfamilies Amiseginae and Loboscelidiinae are believed to be obligate parasitoids of phasmid eggs (Krombein 1983), and as such, they have potential for the biological control of phasmid outbreaks and infestations. Our knowledge of host preference and host specificity is currently insufficient, and further work is required to establish the feasibility of these wasps as control agents.

The rate at which this knowledge is acquired could be increased if studies were made by the large number of people interested in phasmids, from pet keepers through to professional taxonomists. It is hoped that by making knowledge of these enigmatic wasp species freely available online that further research on these groups is encouraged, both by hymenopterists and phasmatologists.

## General description

### Purpose

The last comprehensive species catalogue of the Amiseginae and Loboscelidiinae was published in Kimsey and Bohart (1991). The database presented here draws heavily on this work, and brings it up to date through the incorporation of information from subsequent publications and online presentation. Summary statistics are provided in Table 1.

The publication of this data paper is intended not only to publicise the online database, but also to provide a convenient method of citation. The Phasmida Species File Online (Brock 2015) has been cited as a website in multiple fashions with varying dates of ‘publication’. A data paper (Chavan and Penev 2011) provides a single method of citation, complete with a Digital Object Identifier (DOI).

The database is online at http://chrysididae.speciesfile.org.

### Additional information

The Chrysididae SpeciesFile was created by entering the Amiseginae and Loboscelidiinae data provided in Kimsey and Bohart (1991) to create an initial taxonomic hierarchy. Original references were then checked for additional information, and Google Scholar was used to identify publications since 1991. The references of all publications used were checked for additional sources of information. Notes next to references that contain keys and images are made on individual species pages.

## Geographic coverage

### Description

**Figure 1.**
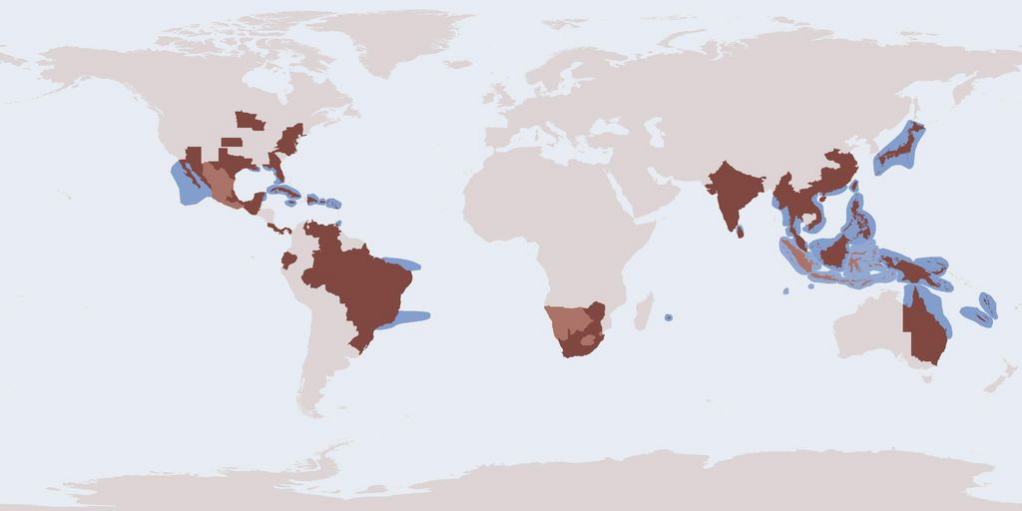
Distribution of the Amiseginae and Loboscelidinae, generated by the Chrysididae Species File.

The database has global coverage of the two subfamilies, the combined distribution of all taxa covered is shown in Fig. 1

## Taxonomic coverage

### Description

**Figure 2.**
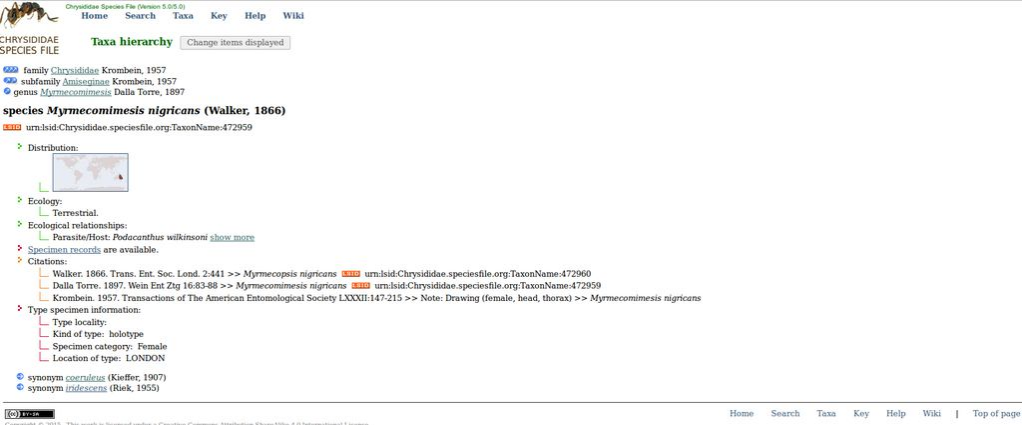
Taxon page for *Myrmecomimesis nigricans* showing higher classification, known hosts, a link to specimen records, references, details of the primary type and synonymy.

The database contains records of all known valid species of Amiseginae and Loboscelidiinae, with full details of synonymy within these subfamilies, both recent and fossil. Full details are given for all type specimens, type species and the first usage of family-group names Fig. 2. Additional specimens from collections have been added from the literature to provide fuller coverage of biogeography.

The Chrysididae Species File can generate distribution maps for any of the taxa it contains. An example showing the overall distribution of the Amiseginae and Loboscelidiinae is shown in Fig. 1

### Taxa included

**Table:** 

Rank	Scientific Name	Common Name
subfamily	Amiseginae	
subfamily	Loboscelidiinae	

## Traits coverage

All known host associations are included and referenced. These are displayed on the relevant taxon page (Fig. 2). A complete list can be obtained by using the 'Other taxon in an ecological relationship' option on the 'Search' page.

## Usage rights

### Use license

Other

### IP rights notes

This work is licensed under a Creative Commons Attribution-ShareAlike 4.0 International License

## Data resources

### Data package title

Bibliography of Amiseginae and Loboscelidiinae

### Number of data sets

1

### Data set 1.

#### Data set name

Bibliography of Amiseginae and Loboscelidiinae

#### Data format

plain text

#### Number of columns

1

#### Download URL

http://dx.doi.org/10.5519/0052040

#### Description

List of works featuring the subfamilies Amiseginae and Loboscelidiinae

**Data set 1.** 

Column label	Column description
none	Bibliographic citation

## Additional information

### Data Sharing

The use of the SpeciesFile software allows for sharing taxonomic data with Species2000/Catalogue of Life, and specimen data with the Global Biodiversity Informatics Facility (GBIF). This is achieved through the DarwinCore Archive format, which has become the standard format for sharing biodiversity realted datasets (e.g. Baker et al. 2014) and build on the DarwinCore standard (Wieczorek et al. 2012). The Chrysididae Species File DarwinCore Archive can be downloaded from Baker 2015b.

### Updating

The species file will be maintained by the author, who will gratefully receive any new papers on these subfamilies via e-mail. At present there are no plans to include the other subfamilies of Chrysididae (Chrysidinae and Cleptinae) unless appropriate editors volunteer their services.

## Figures and Tables

**Table 1.** Overview statisitics for the Chrysididae Species File.

Unique authors	46
Unique references	69
Specimen depositories	36
Speciem records	347 (178 unique taxa)
Genera	51 (36 valid)
Species	197 (187 valid)
Names at all ranks	252 (225 valid)
